# An *in vivo* Calcium Imaging Approach for the Identification of Cell-Type Specific Patterns in the Developing Cortex

**DOI:** 10.3389/fncir.2021.747724

**Published:** 2021-10-05

**Authors:** Alicia Che, Natalia V. De Marco García

**Affiliations:** Center for Neurogenetics, Brain and Mind Research Institute, Weill Cornell Medicine, New York, NY, United States

**Keywords:** *in vivo* imaging, 2-photon calcium imaging, development, interneuron, spontaneous activity

## Abstract

Neuronal activity profoundly shapes the maturation of developing neurons. However, technical limitations have hampered the ability to capture the progression of activity patterns in genetically defined neuronal populations. This task is particularly daunting given the substantial diversity of pyramidal cells and interneurons in the neocortex. A hallmark in the development of this neuronal diversity is the participation in network activity that regulates circuit assembly. Here, we describe detailed methodology on imaging neuronal cohorts longitudinally throughout postnatal stages in the mouse somatosensory cortex. To capture neuronal activity, we expressed the genetically encoded calcium sensor GCaMP6s in three distinct interneuron populations, the 5HT3aR-expressing layer 1 (L1) interneurons, SST interneurons, and VIP interneurons. We performed cranial window surgeries as early as postnatal day (P) 5 and imaged the same cohort of neurons in un-anesthetized mice from P6 to P36. This Longitudinal two-photon imaging preparation allows the activity of single neurons to be tracked throughout development as well as plasticity induced by sensory experience and learning, opening up avenues of research to answer fundamental questions in neural development *in vivo*.

## Introduction

Early spontaneous activity is fundamental for the development of neurons, regulating cellular and synaptic processes. However, the highly correlated activity patterns observed in development would interfere with stimulus-driven sensory coding in the mature cortex (Clancy et al., 2015). Therefore, immature neural networks must undergo dramatic transformations during the early postnatal period, particularly from the first to second postnatal week. In a matter of a few days immediately before eye opening and at the onset of active whisking, synchronous large-scale calcium waves transition into sparser patterns dominated by decorrelated firing of cortical neurons (Golshani et al., 2009; Rochefort et al., 2009; Allene and Cossart, 2010; Colonnese et al., 2010; Che et al., 2018; Pires et al., 2021). At the network level, this desynchronization is thought to be internally mediated and independent from sensory inputs (Golshani et al., 2009; Rochefort et al., 2009; Colonnese et al., 2010). Interestingly, this period of desynchronization coincides with the critical period during which cortical interneurons go through rapid maturation and integration (Batista-Brito and Fishell, 2009; Lim et al., 2018). Unlike pyramidal cells, the activity of many interneuron subtypes during this stage is heavily influenced by direct sensory inputs (García et al., 2015; Che et al., 2018; Modol et al., 2020). Due to limitations in the targeting of specific interneuron subtypes, and the fact that they are largely outnumbered by pyramidal cells, it remains unclear if interneuron populations also undergo similar desynchronization. Moreover, their activity patterns and function during this stage are not well understood.

Efforts to study how interneuron subtypes contribute to circuit development at a cell-type specific level have been hampered, in part, by technical challenges in tracking neuronal activity longitudinally throughout different developmental stages. While studies in adolescent and adult mice successfully utilize long-term *in vivo* imaging to survey circuit changes underlying specific behaviors (Peron et al., 2015; Pinto and Dan, 2015; Pattwell et al., 2016), most *in vivo* studies in neonatal mice are limited to acute experiments (Garaschuk et al., 2000; Golshani et al., 2009; Minlebaev et al., 2011; Yang et al., 2013). Although these approaches provided great insight into our understanding of network patterns and their development, they lack cell-type specificity, and do not allow the assessment of long-term changes occurring before, during, and after a given developmental event or manipulation. Longitudinal imaging in mouse pups is challenging due to the following factors: (1) Anesthesia and cranial window surgery can be technically difficult at neonatal age; (2) Surgery and implantation could discourage maternal care or even lead to cannibalization of the pups; (3) Size and weight of the implanted head plate could impede pups’ feeding and mobilization; (4) The implant could affect head growth and/or general development of the pup; (5) Quality of the cranial window declines over time. To overcome these challenges, we devised modified head plates, a surgery procedure and a customized care protocol, which effectively allowed for long-term calcium imaging of the same mouse from neonatal stage (P5 to 6) into young adulthood (P35). This facilitates the tracking of neuronal activity in the same cohort of neurons as development progresses.

Here we describe in detail a cranial window preparation and head plate implant surgery for longitudinal imaging of interneuron subtypes over the somatosensory cortex, as we have previously published (Che et al., 2018; Duan et al., 2020). In particular, we provide a description and rationale for the modifications we have made for young animals to aid any further adjustments the experimenters may desire to make to suit their particular needs. As this surgical procedure during the first postnatal week and post-surgery long-term survival can be challenging, we aim to provide alternative surgical options from those previously described (He et al., 2018), and additional discussions on maternal and long-term care considerations. We provide examples of longitudinal 2-photon calcium imaging from three different interneuron subtypes, 5HT3aR-expressing L1 interneurons, SST interneurons, and VIP interneurons. Lastly, we discussed possible applications of this technique in addressing central questions on cortical development.

## Materials and Methods

### Use of Transgenic Mice for GCaMP Expression

Animal handling and experimentation were performed in accordance with United States National Institutes of Health and Weill Cornell Medical College Institutional Animal Care and Use Commission. Mice of both sexes were used and housed in a controlled environment on a 12 h light/dark cycle (6 am to 6 pm) with food and water *ad libitum*. In the current study, *5HT3aR.Cre*, *SST*^Cre^, and *VIP*^Cre^ mice were crossed with *RCL-GCaMP6s* mice (Ai96, Jackson Laboratories, 024106). We have previously shown that the GCaMP6s expression in these crosses does not cause maturation, migration or survival defects (Che et al., 2018). This is consistent with reports that while some genetic crosses, especially those using intersectional strategies, produce mice that exhibit seizure-like activity, no aberrant activity is observed for crosses with the Ai96 line (Steinmetz et al., 2017). It should be noted that development and activity should be assessed in a case-by-case basis when other lines are used. It is also feasible to use a combination of genetically encoded reporter, such as Ai9, in combination with a more targeted injection of virally encoded GCaMP of choice to avoid potential aberrant activity, to increase the number of populations labeled, and/or to deliver genetic manipulations. We and others have previously used this method successfully in neonatal mice (Che et al., 2018; He et al., 2018; Duan et al., 2020).

### Head Plate Implant

**FIGURE 1:**
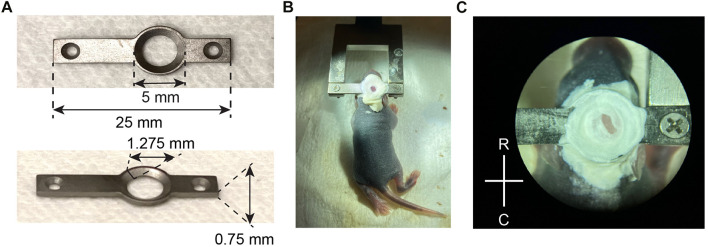
Head plate design and attachment. **(A)** Design and key measurements for the titanium head plate used in neonatal mice. **(B)** A P6 mouse pup stabilized for surgery, after the head plate was attached with Metabond and screwed into a fork-shaped holder with two 1/8” screws. **(C)** A close-up view of the exposed skull after the head plate was attached, before the craniotomy was made. R, rostral; C, caudal.

For mice aged P7 and under, hypothermia was found to be the most effective method of anesthesia. Mouse pups were submerged in ice for 5 min, and excess water was wiped dry from the pup upon removal from the ice. It is essential that the ice is fresh, not melting icy water, to avoid drowning and excess cold. The head was then scrubbed with alternating solutions of 70% ethanol and 10% povidone-iodine (Betadine^®^). All surgical surfaces and instruments used were sterilized. Local anesthesia was provided by infiltrating bupivacaine (Marcaine, 0.25–0.5% solution) into the tissue adjacent to the intended incision lines. Subsequently, the scalp was removed, and care was taken not to make incisions too close to the eye and ear regions where capillary blood vessels are abundant in the skin. The periosteum covering the skull was removed by scrubbing gently with a cotton-tipped applicator. The skull is soft and pliable at this age, so care should be taken to avoid excess force, which could result in damage and bleeding of the brain. Once the skull was cleaned and dry, the location of the somatosensory cortex was determined by stereotaxic coordinates [centered at (from Lambda): AP 1.6–2.2, ML 1.8–2.0, DV 0.1–0.3] and marked by a Sharpie to visually guide the placement of the head plate. The coordinates were empirically tested and adjusted for the age of the pups. The location of the cranial window was also confirmed *post hoc* for each mouse. Using a previous design as a blueprint (Pattwell et al., 2016), we devised a custom-made light-weight titanium head plate (average ∼280 mg) so that it does not impede on the development and mobility of the pup, and yet strong enough for unanesthetized imaging sessions (Figure 1A). The small opening design of the head plate also minimizes the contact area on the skull so it does not affect skull growth (no gross malformation was detectable after the head plate was removed at P35–P40). The “wings” of the head plate are also long enough to clear the sides of the head as it grows. The head plate was then adhered to the skull using a veterinary adhesive (Metabond^®^). It should be noted that a generous amount of Metabond^®^ should be applied to ensure a strong hold between the skull and the head plate, as well as covering any exposed skull, but care should be taken not to cover excessive skin, ears or eyes with Metabond^®^ (Figures 1B,C). After the head plate was attached and Metabond^®^ cured, for about 5–10 min, the mouse was placed back on ice for 2–3 min if tail-pinch reflex has returned.

### Cranial Window Surgery

**FIGURE 2:**
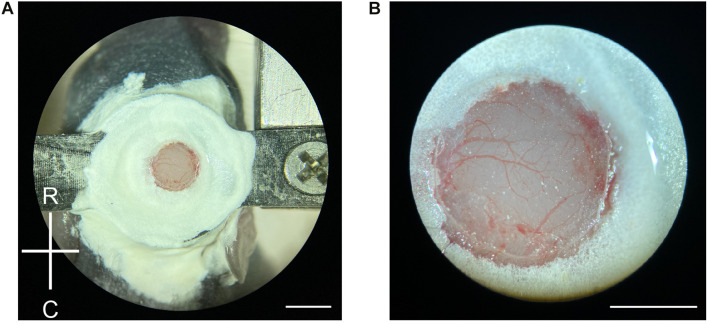
Craniotomy in neonatal mice. **(A)** Demonstration of a newly made craniotomy on a P6 mouse. The cranial window is about the same size as the 2-mm diameter coverslip. R, rostral; C, caudal. Scale = 2 mm. **(B)** A close-up view of the craniotomy after the completion of the surgery. Blood vessels are clearly visible with minimal bruising. Scale = 1 mm.

The head plate was screwed to a custom-made holder. Short (1/8”) screws were used so that they did not protrude from the bottom of the head plate to accommodate head growth. The craniotomy was performed using the sharp edge of a sterile 16G or 18G syringe needle. The skull is pliable and thin under the age of p12, and while others have used small dental drills for mice as young as P8 (He et al., 2018), we found the needle approach to be much more effective and less likely to cause damage to the brain. A circle 2–3 mm in diameter was gently etched at the center of the head plate by repeatedly thinning the skull around the circumference with the needle. After the circle was sufficiently thinned, a small cut through the skull was made at the 4 o’clock position (8 o’clock if left-handed), and a pair of fine-tipped forceps was used to lift off the skull piece at the incision. This should be attempted while the skull was submerged in sterile PBS. Care was taken to preserve the dura and the surface blood vessels, as they are easily damaged at this age. If bleeding occurs, surgical eye spears, and Gelfoam sponge were used to stop minor bleeding. After achieving hemostasis, excess PBS was removed from the craniotomy and the dura was dried. A 2 or 3 mm diameter, 0.1 mm coverslip was lowered on top of the brain, and warm (37°C) 1% low-melt agarose was applied to the perimeter of the craniotomy to seal. The coverslip was then fixed to the skull using veterinary adhesives, first Vetbond^®^ (applied with a 10 μl pipette tip), then Metabond^®^ (Figures 2A,B). We apply the three-step agarose-Vetbond^®^ -Metabond^®^ sealing approach for the following reasons: (1) The agarose fills any gaps that might exist around the edge of the coverslip due to brain curvature and acts as a barrier between the brain and the adhesives. (2) Vetbond^®^ seals and hold down the coverslip. (3) Lastly, Metabond^®^ is applied to further strengthen the coverslip and cover any exposed skull to ensure that the cranial window is secure for longitudinal imaging. Note that Metabond should be applied only after Vetbond^®^ has dried completely.

For an expert experimenter, the cranial window surgery should take 35–40 min, including time for setup and anesthesia. It could take up to 1 h for a novice experimenter, which would likely require additional anesthesia (hyperthermia) during the procedure and could impact window quality and survival.

### Recovery and Husbandry Considerations

After the surgery, the pup was placed on a heating pad with bedding from the home cage for recovery. The scent of bedding masks the odor of adhesives and lowers the chance of pup rejection by the dam. Chance of pup survival and higher quality of maternal care can be achieved by the following practices: (1) If possible, use experienced breeders. (2) Use pups from a harem breeding cage (one male, two females). The additional female, in particular if an experienced breeder, improves pup survival. (3) Remove the male from the cage prior to surgery. (4) Remove excess pups from a large litter so that pups with head plate implant do not need to compete for access to milk and care. (5) High level of cleanliness, pathogen-free environment, and consistent care in the animal care facility is key in reducing maternal stress and increasing pup survival.

### Estimated Success Rate and Modifications for Surgery on Pups in Second Postnatal Week

For a novice experimenter, we estimate that the success rate for the placement of cranial windows with clear optical access over all the surgeries performed on P5–P7 mouse pups is about 50%. In about 60–80% of those pups, the quality of the window will permit imaging for up to a week from the day of the surgery. For an expert experimenter, the success rate of the cranial window surgery can reach 80–90%, and 90% of those pups would likely be suited for week-long imaging. Achieving high quality month-long longitudinal imaging is difficult for novice experimenters, but experts show a 30–50% success rate. The Success rate of long-term imaging can be improved by performing cranial window surgeries on older pups if the early postnatal period is not of interest. For pups older than P7, isoflurane should be used for anesthesia instead of hypothermia. Eye ointment should be applied on pups older than P12 during surgery and imaging sessions. After P10, craniotomy can be made easier by thinning the perimeter with a dental drill instead of a needle.

### *In vivo* Two-Photon Calcium Imaging

Mouse pups can be imaged 3–4 h after the cranial surgery on the same day and on subsequent days. Imaging was performed on un-anesthetized mouse pups while the head was stabilized to a holder using two 1/8” set screws. The pup was kept on a 37°C heating pad, and cotton balls or bedding were placed loosely around the pup for comfort. During imaging sessions, mouse pups should spend most of the time in a quiet resting state, interrupted by occasional limb or tail twitches. If the pups are in distress (struggling, vocalizing), most commonly the height of the head plate needs to be adjusted so that the pup rests comfortably. Scent of the home cage bedding decreases the stress level in pups. Imaging sessions should be limited to no longer than 30 min at a time. In our experiments, a FluoView FVMPE-RS multiphoton imaging system (Olympus) was used for detecting calcium events in mouse pups. A Mai Tai^®^ DeepSee Ti:Sapphire laser (Spectra-physics) was tuned to 920 nm for GCaMP6s excitation and the total laser power delivered to the brain was less than 60 mW. A 25X (1.05 NA) water immersion lens (Olympus) was used for imaging. The system was controlled by FV30S-SW software (Olympus). Image frames of 509 μm × 509 μm (512 × 512 pixels) were recorded using a Galvano scanner at a rate of 1 Hz for all *SST.GCaMP6s* and *VIP.GCaMP6s* longitudinal imaging, and using a resonance scanner at 5 Hz (66 ms per frame, averages every three frames) for all *5HT3aR.GCaMP6s* longitudinal imaging.

**FIGURE 3:**
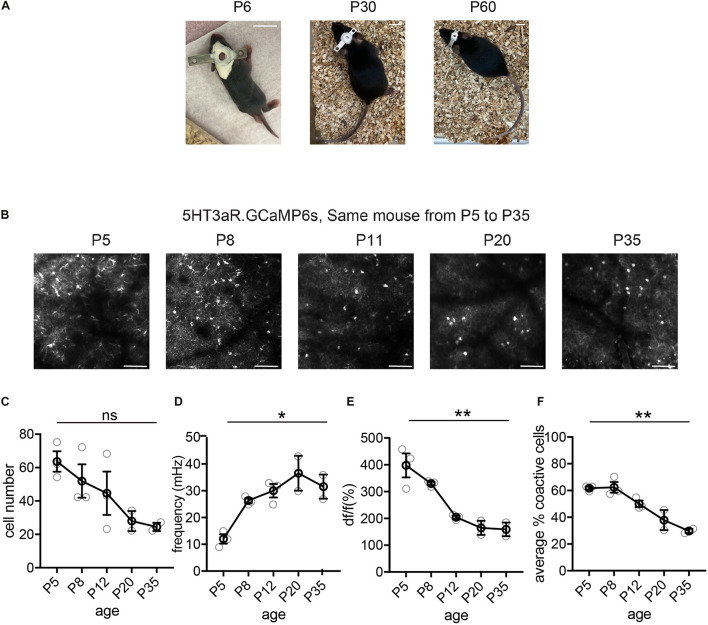
Longitudinal imaging of L1 5HT3aR interneurons. **(A)** Representative image of a mouse with head plate implant at P6, P30, and P60. Scale = 10 mm. **(B)** Representative two-photon field of views (FOV)s from a 5HT3aR.GCaMP6s mouse images at P5, P8, P11, P20, and P35. Scale = 100 μm. **(C)** Quantification of the number of active cells in a FOV at P5, P8, P11, P20, and P35. Kruskal–Wallis test, *p* = 0.086. *n* = 3 FOVs for P5, P8, and P11, *n* = 2 FOVs for P20 and P35, from three different longitudinally imaged animals. **(D)** Quantification of the average event frequency for each cell. *p = 0.034. **(E)** Quantification of the average event amplitude for each cell. **p = 0.013. **(F)** Quantification of the average percentage of coactive cells. **p = 0.015. Black circles indicate mean from all videos; gray circles indicate individual videos; error bars indicate s.e.m.

For chronic imaging, mouse pups were returned to their home cage with the dam at the end of the imaging session. The conditions of the animal, head plate, and cranial windows were monitored daily. Subsequent imaging sessions took place every 24 or 48 h, until the animal was sacrificed. With the exception of P5 analysis for which we performed cranial window surgery and imaging on the same day between 2 pm and 5 pm (at least 3–4 h post-surgery), our other imaging sessions always occur between 9 am and 12 pm. The presence of the head plate did not appear to impair feeding, grooming, or interactions between the pup and its mother as well as littermates. Body size of head-implanted animals did not differ from that of their littermates, nor did these animals display any gross developmental impairment (Figure 3A). During each imaging session, the same FOV was located using blood vessels and the borders of the cranial window as landmarks. Imaging depths were adjusted as the animal matured to ensure that the same cohort of neurons was imaged. This was achieved by finding a depth that represent the most number of matched cells in the same FOV. The depth of the same FOV can increase up to 200 μm from P6 to P36. In some cases, the same neurons were tracked daily during the entire span of the experiment. We have previously tracked all cells in a FOV to determine the fate of each individual cell from P6 to P8 (Duan et al., 2020). To do this, we identified the same region of the cortex using the borders of the cranial window and vascular landmarks. To image MGE-derived interneurons, we use the vascular landmarks present both on the surface and deeper in the brain which are also delineated by TdTomato in *Lhx6.Ai9* mice (Lhx6 is expressed by both interneurons and endothelial cells). This can also be achieved by using tail vein injection of TRITC-Dextran (50 μl at 5 mg/ml). After the FOV was confirmed to match the FOV from the previous imaging session, a z-stack of 50 μm above and 50 μm below the FOV at 4 μm interval was taken at 1040 nm (red). In the red channel, each Ai9-labeled cell was located (or deemed dead) based on the matching video from the previous session. To confirm a cell had indeed gone through apoptosis, we compared the max projection of the z-stacks from the two sessions and manually searched in the z-stack, as it is common to see cells moving out of the imaging plane due to the growth of the brain, in particular between P6 and P10. After the last imaging session, the mice were sacrificed and the location of the cranial window was confirmed by topically placing Dextran (Thermo Fisher Scientific) or DAPI (Thermo Fisher Scientific) over the window after removing the coverslip.

### Imaging Data Processing and Analysis

The imaging data processing method used in this study has been described in our previous publications (Che et al., 2018; Duan et al., 2020). Briefly, processing and calcium-signal detection were carried out using CalciumDX software routines written in MATLAB (Mathworks) (Ackman et al., 2012)^1^. Movies were first motion corrected using the Image Stabilizer Plugin for NIH Image J^2^. To ensure imaging data were collected from un-anesthetized neonatal mice that are quietly resting with only cardiopulmonary or myoclonic twitch movements, recordings containing segments of large motion artifacts were excluded from the analysis. Movies of neuronal activity consisting of time series images were collected as *t*-stacks. For each movie, cell contours were semi-automatically detected in the *t*-stack projections of the average intensity image using an edge-detection algorithm, and calcium signals were measured as the average intensity inside each cell contour (Crépel et al., 2007; Allène et al., 2008; Ackman et al., 2012). The Δ*F/F* signal was then calculated for every contour/grid in each frame. Calcium transients were identified using automatic detection algorithms. Briefly, baseline de-trending was performed by applying a high pass filter, and a temporal sliding window with a length of 3 frames was used to determine baseline average (Dombeck et al., 2007). The threshold of detection was set as 2 standard deviations above the baseline average plus 2 standard deviations above the derivative of the signal (local maxima). The onset of calcium events was set as the first frame in the rising phase of the calcium transient, and the offset was set as the half-amplitude of the decay time. Active cells are neurons with at least one detected calcium event during the entire movie. Neuropil correction was performed by subtracting signal in a shell around the detected soma from the soma signal.

Data were analyzed using custom routines written in MATLAB as previously described (Allène et al., 2008; Che et al., 2018; Duan et al., 2020). In this study, we characterized calcium activity characteristics for individual neurons by measuring average calcium transient event frequency and event amplitude for each cell, detected as described above. To quantify the percentage of coactive cells, event histograms that plotted the percentage of cells active for each frame were first constructed. Peaks of co-active cells in the histogram were measured and then averaged to determine the percentage of coactive cells in a given video. We chose to quantify the percentage of coactive cells here instead of the network analyses we have previously used, as network events cannot be easily defined in animals after the second postnatal week without time-locked stimulation or cue and without information on the behavioral states of the animals. Percentage of coactive cells gives a broader measure of the spontaneous activity level and synchronicity of the activation across developmental stages.

Statistical analyses were performed on the mean of all movies from multiple animals for each developmental stage. If more than one movie from the same mouse were used for a given age, these movies corresponded to non-overlapping FOVs and therefore consisted of different cell populations. We used Kruskal–Wallis test to evaluate statistical significance, as indicated in the figure legends.

## Results

We performed cranial surgery and window implants on P5–7 mouse pups, and demonstrate here examples of longitudinal imaging and basic event characteristics of three distinct interneuron subtypes, the 5HT3aR L1 interneurons, SST interneurons, and VIP interneurons. Interestingly, these three populations show distinct developmental trajectories in network characteristics from as early as the first postnatal week. In the L1 interneuron population, the trend for the number of detected cells in a FOV is to decrease from P5 to P35 (Figures 3B,C), likely due to the expansion of the brain and the regrowth of the skull bones. The frequency of the detected events, on the other hand, increased during the first month, with changes particularly dramatic during the first two postnatal weeks (P5–P12, Figure 3D). We also observed a marked decrease in event amplitude during this period (Figure 3E). We have previously performed whole-cell patch clamp recording on early postnatal neurons during spontaneous network events, and found that they largely displayed bursting firing (Che et al., 2018). The increase in frequency and decrease in amplitude in calcium events likely reflect the transition from firing in bursts during the early postnatal stage, which results in longer and larger calcium events, to more discrete single action potential firing in mature animals. Consistent with our previous report, the synchronicity of L1 interneuron activation, as measured by the average percentage of coactive cells, decreased significantly from the first to the second postnatal week (Che et al., 2018), and continued to decrease to P35 (Figure 3F).

**FIGURE 4:**
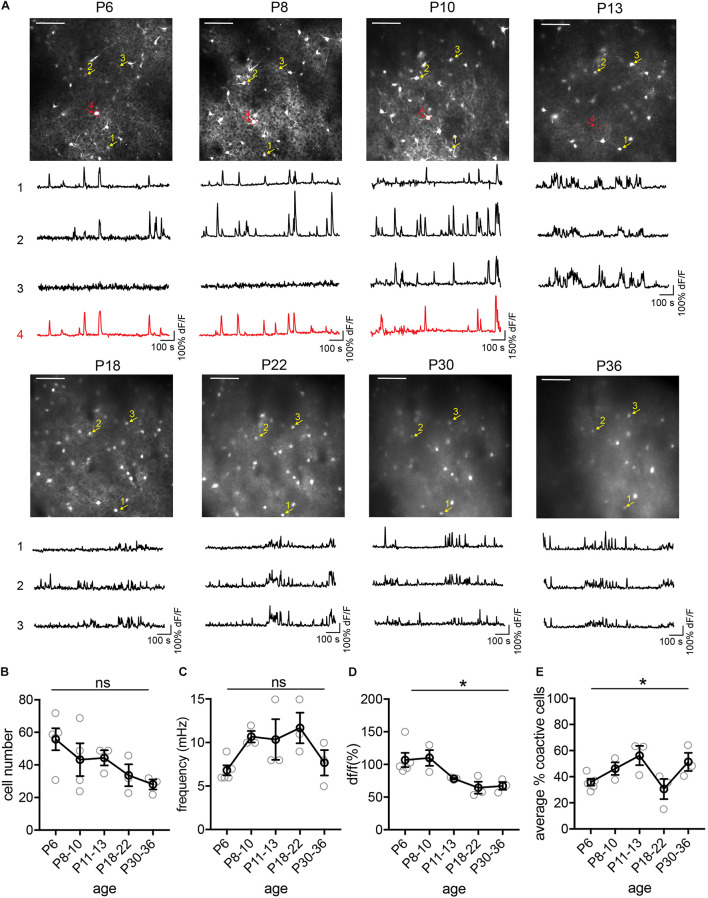
Longitudinal imaging of SST interneurons. **(A)** The same two-photon FOV from a SST.GCaMP6s mouse at P6, P8, P10, P13, P18, P22, P30, and P36. Scale = 100 μn. The same example cells across different ages are indicated by numbered arrows. The yellow ones survived from P6 to P30, while the red one died between P10 and P13. Corresponding raw ΔF/F traces are shown below each FOV. **(B)** Quantification of the number of active cells in a FOV at P6, P8–10, P18–22, and P30–36. Kruskal–Wallis test, *p* = 0.12. *n* = 5 FOVs for P6, 4 FOVs for P8–10, 3 FOVs for P11–13, P18–22, and P30–36, from two longitudinally imaged animals. **(C)** Quantification of the average event frequency for each cell, *p* = 0.07. **(D)** Quantification of the average event amplitude for each cell. *p = 0.014. **(E)** Quantification of the average percentage of coactive cells. *p = 0.041. Black circles indicate mean from all videos; gray circles indicate individual videos; error bars indicate s.e.m.

**FIGURE 5:**
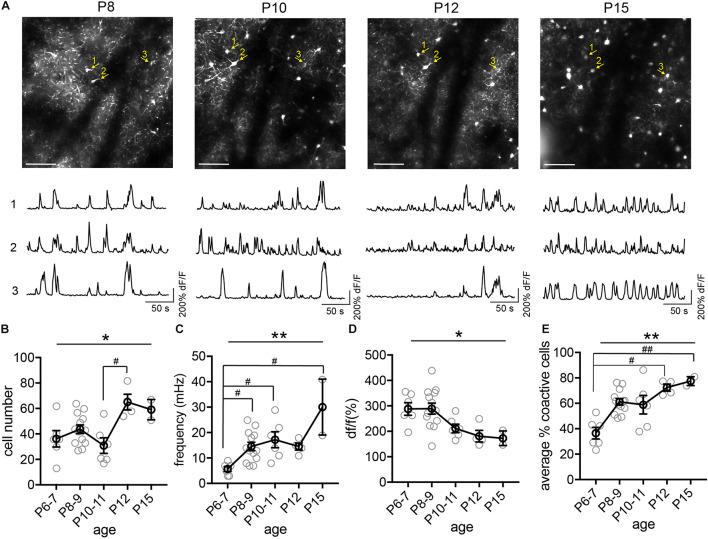
Longitudinal imaging of VIP interneurons. **(A)** The same two-photon FOV from a VIP.GCaMP6s mouse at P8, P10, P12, and P15. Scale = 100 μm. The same example cells across different ages are indicated by numbered yellow arrows. Corresponding raw ΔF/F traces are shown below each FOV. **(B)** Quantification of the number of active cells in a FOV at P6–7, P8–9, P10–11, P12, and P15. Kruskal–Wallis test, *p = 0.016. *n* = 6 FOVs for P6–7, 13 FOVs for P8–9, 6 FOVs for P10–11, 4 FOVs for P12, and 2 FOVs for P15, from five animals. **(C)** Quantification of the average event frequency for each cell. **p = 0.0061. **(D)** Quantification of the average event amplitude for each cell. *p = 0.016. **(E)** Quantification of the average percentage of coactive cells. **p = 0.028. Black circles indicate mean from all videos; gray circles indicate individual videos; error bars indicate s.e.m.

Next, we tracked the activity of the same cohorts of SST interneurons from P6 to P36, identifying the same neurons throughout this period (Figure 4). We found that during the second postnatal week, SST interneurons went through the most dramatic changes in their activity, location, and survival (Figure 4A). In particular, longitudinal imaging allows for the identification and comparison between SST interneurons that go through apoptosis and those who survive, as we have previously analyzed in more detail (Figure 4A, cell 4; Duan et al., 2020). Notably, unlike L1 interneurons and pyramidal cells (Golshani et al., 2009; Che et al., 2018), SST interneurons did not show marked developmental desynchronization before P13 – the percentage of coactive cells increased during the second postnatal week (Figure 4E). In the VIP interneuron population (Figure 5), we observed a significant increase in the number of detected cells between P10 and P12. This may reflect an increase in the number of VIP interneurons that become more active, perhaps due to an increase in cell activation around the onset of eye opening and active whisking, or increased synaptic connections in this cell type during the second postnatal week. In addition, the percentage of coactive cells also increased significantly from P6 to P15 in the VIP population (Figure 5E). It is worth noting that while 5HT3aR L1 interneurons exhibit developmental desynchronization on the network level as do pyramidal cells during the second postnatal week, other interneuron populations, including SST and VIP interneurons, do not. It has been reported previously that SST and VIP interneurons desynchronize after P21 (Kastli et al., 2020), suggesting that these populations may increase in synchrony during the second postnatal week but desynchronize later. The increase in event frequency and decrease in event amplitude, on the other hand, appear to be consistent across interneuron subtypes.

## Discussion

We presented here detailed methods on performing craniotomy and cranial window implant on neonatal mice, as well as considerations on long-term 2-photon imaging through development. We have previously used these methods to elucidate early spontaneous activity of interneurons and pyramidal cells, and how changes in interneuron network activity lead to disruptions in circuit formation (Che et al., 2018; Duan et al., 2020). Here, we demonstrated the use of long-term imaging by measuring the spontaneous activity of three distinct interneuronal subtypes, the 5HT3aR L1 interneurons, SST interneurons and VIP interneurons using transgenic mice expressing GCaMP6s driven by subtype-specific *Cre* drivers. We were able to track the activity of the same cohort of neurons, as well as the same neurons in some instances, through the first postnatal week up to P36 using the same cranial window. While other longitudinal imaging methods have been described previously (Mizuno et al., 2014; He et al., 2018), we describe here the use of transgenic mice to target and longitudinally image different cell types from as early as P5. Given the challenges presented in long-term imaging in developing mice, including performing cranial window surgeries during neonatal stage, ensuring survival, and preserving imaging quality over time, our methods will provide experimenters with technical alternatives and additional options in optimizing their own procedures to suit their experimental needs. The successful implementation of longitudinal imaging method over development opens up new avenues for research that requires the understanding of developmental trajectory of neuronal activity, function and morphology.

Developmental desynchronization, or the transition from bursting to continuous firing, has been previously documented for different sensory cortices, in particular the visual and somatosensory cortex (Golshani et al., 2009; Colonnese et al., 2010; He et al., 2018). However, neuronal synchronicity has only been described at a network level. As pyramidal cells vastly outnumber interneurons, this desynchronization is mostly attributed to properties of pyramidal cells and the excitatory network. The underlying mechanisms for this desynchronization are likely complex – developmental changes in intrinsic cell properties, expression of ion channels and receptors in cortical pyramidal cells, as well as transitions in thalamocortical and neuromodulatory inputs have all been proposed to influence network synchrony. It remained unclear whether different cell types go through the same process, and if so, whether they are controlled by excitatory inputs or require cell-type specific internal changes. We found that developmental desynchronization is not a universal phenomenon in all cell types. The developmental trajectory in network patterns diverge between interneuron subtypes as well – while L1 5HT3aR interneurons desynchronize rapidly from the first to second postnatal week much like neighboring pyramidal cells, SST and VIP interneurons do not show apparent desynchronization. Our results are consistent with previous finding that low-threshold spiking SST interneurons abruptly increase in spike synchronization between P12 and P15, influenced by rapid and concurrent changes in several intrinsic properties and synaptic connections (Long et al., 2005; Urban-Ciecko and Barth, 2016). This synchronized activity in SST neurons is thought to be important for the onset of exploratory whisking and the related somatosensory processing in pups (Long et al., 2005). While pyramidal cells may drive network patterns and interneuron activation during the first postnatal week (Duan et al., 2020), our findings here suggest that different neuronal types diverge in their activity patterns, and that the underlying mechanisms for the change in their synchronization are cell-type specific. Future studies utilizing the longitudinal imaging method described here could shed light on the mechanisms and the functions of these developmental transitions in different cell types.

It is also important to consider the limitations of our longitudinal imaging method in experimental design. First, the earliest stage for long-term cranial window implantation without causing any developmental disruptions in our hands is P5. While we have successfully performed P4 and earlier cranial window surgeries followed by acute imaging using a similar method as the one used for P5 onward, we find that P0–P4 mice who have undergone head plate implant appear smaller in size and weigh less compared to their littermates during the first 2–3 postnatal weeks. Therefore, while they catch up in size eventually, the potential developmental impact of the surgical manipulation prevented us from analyzing mice younger than P5. This is likely due to the fact that the head plates are too heavy for the young pups and this may impede proper feeding. Reducing the weight and size of the head plate further, however, affects imaging stability of juvenile mice. Experimenters who wish to cover ages younger than P5 but do not wish to image pass the second or third postnatal week could consider further reducing the size of the head plate design described here to suit their specific needs. Second, skull regrowth is significant between P5 and P35. A large cranial window is required when performing long-term imaging covering such extended developmental period, as the skull will grow back to leave only one or a partial FOV clear for imaging. Preferably, the cranial window should be replaced before P30, and excess skull can be removed to enlarge the imaging area. This should extend the imaging age to P40 and beyond, opening the possibilities for more experimental designs involving mature animals, such as behavioral testing and rescue experiments. Lastly, although the same cohort of neurons and some individual neurons can be followed in the same FOV, it can be challenging to follow every neuron from P5 to P36. Interneurons are still migrating to their final locations at the end of the first postnatal week, and the density of pyramidal cells makes them difficult to track and differentiate from one another. In addition, cells in the areas close to the borders of the FOV can disappear out of the FOV due to rapid brain growth during the first two postnatal weeks. We lose 10–15% area along the borders if imaging for over a week from the first to the second postnatal week and those cells cannot be tracked. We have previously tracked every Lhx6-expressing neuron in a FOV from P6 to P8, using Ai9 expression and z-stack to verify the location of each neuron (Duan et al., 2020). Tracking individual neurons for longer periods of time may require sparse labeling with viral infection to aid with identification and tracking of the neurons.

## Conclusion

Longitudinal methods to image the brain *in vivo* with single-cell resolution combined with genetic tools offer the ability to examine the maturation, activity, and function of different cell populations through critical periods of development. Here we provide a detailed description on the surgical procedures and considerations to improve post-op survival and improve success rate, as the surgery has been reported to be very challenging in neonatal mice. This is important particularly in experiments involving complex genetic crosses and additional viral injections, as the number of pups with the desirable genotype might be already small in a given litter. The ability to assess activity in the same cohort of neurons from the first postnatal week to P36 and beyond will provide the means to assess morphological and functional changes before, during and after learning, drug treatment, and onsets of pathology such as epilepsy.

## Data Availability Statement

All data generated and analyzed for this study area will be made available upon request to the corresponding author.

## Ethics Statement

The animal study was reviewed and approved by the Weill Cornell Medical College Institutional Animal Care and Use Commission.

## Author Contributions

AC and ND designed the research and wrote the manuscript. AC performed the experiments and analyzed the data. Both authors contributed to the article and approved the submitted version.

## Conflict of Interest

The authors declare that the research was conducted in the absence of any commercial or financial relationships that could be construed as a potential conflict of interest.

## Publisher’s Note

All claims expressed in this article are solely those of the authors and do not necessarily represent those of their affiliated organizations, or those of the publisher, the editors and the reviewers. Any product that may be evaluated in this article, or claim that may be made by its manufacturer, is not guaranteed or endorsed by the publisher.

## Footnotes

1
https://github.com/ackman678/CalciumDX

2
http://www.cs.cmu.edu/~kangli/code/Image_Stabilizer.html
